# Straightforward and affordable agroinfiltration with RUBY accelerates RNA silencing research

**DOI:** 10.1007/s11103-024-01463-8

**Published:** 2024-05-19

**Authors:** Midori Tabara, Ayumi Matsumoto, Yuriko Kibayashi, Atsushi Takeda, Kazuki Motomura

**Affiliations:** 1 Ritsumeikan-Global Innovation Research Organization, Ritsumeikan University, Kusatsu, Shiga 525-8577 Japan; 2 Research Organization of Science and Technology, Ritsumeikan University, Kusatsu, Shiga 525-8577 Japan; 3 College of Life Sciences, Ritsumeikan University, Kusatsu, Shiga 525-8577 Japan; 4 Japanese Science and Technology Agency, PRESTO, Kawaguchi, Saitama 332-0012 Japan

**Keywords:** Agroinfiltration, Betalain, *Nicotiana benthamiana*, RNA silencing, RUBY

## Abstract

Transient expression and induction of RNA silencing by agroinfiltration is a fundamental method in plant RNA biology. Here, we introduce a new reporter assay using RUBY, which encodes three key enzymes of the betalain biosynthesis pathway, as a polycistronic mRNA. The red pigmentation conferred by betalains allows visual confirmation of gene expression or silencing levels without tissue disruption, and the silencing levels can be quantitatively measured by absorbance in as little as a few minutes. Infiltration of RUBY in combination with p19, a well-known RNA silencing suppressor, induced a fivefold higher accumulation of betalains at 7 days post infiltration compared to infiltration of RUBY alone. We demonstrated that co-infiltration of RUBY with two RNA silencing inducers, targeting either CYP76AD1 or glycosyltransferase within the RUBY construct, effectively reduces RUBY mRNA and betalain levels, indicating successful RNA silencing. Therefore, compared to conventional reporter assays for RNA silencing, the RUBY-based assay provides a simple and rapid method for quantitative analysis without the need for specialized equipment, making it useful for a wide range of RNA silencing studies.

**Supplementary Information:**

The online version contains supplementary material available at 10.1007/s11103-024-01463-8.

## Key message

Agroinfiltration with RUBY is useful as an RNA silencing reporter system that can be performed inexpensively without complex detection methods.

## Introduction

Agroinfiltration is a transient overexpression method that utilizes the ability of *Agrobacterium tumefaciens* (*Rhizobium radiobacter*) to deliver its T-DNA into infected plants and has contributed widely to advances in plant molecular biology (Gelvin 2003; Tzfira and Citovsky 2006). T-DNA on the binary plasmid vector, when transformed into *Agrobacterium*, can be constructed to express a particular gene *in planta*, depending on the desired research design. Co-infiltration with multiple agrobacterial transformants enables investigation of their product interactions (Llave et al. 2000; Johansen and Carrington 2001). However, agroinfiltration frequently triggers RNA silencing and fails to express foreign genes (Baulcombe 2004).

RNA silencing is a regulatory mechanism that represses the expression of a particular gene in a complementary sequence (Baulcombe 2004). RNA silencing works in both endogenous gene regulation and defense against exogenous genes, including foreign genes introduced by *Agrobacterium* and viral infection. Genes overexpressed by agroinfiltration occasionally trigger the synthesis of double-stranded RNAs from T-DNA-derived transcripts by RNA-dependent RNA polymerase (RDR), which is then processed into small interfering RNA (siRNA) by Dicer. The siRNA is loaded into Argonaute, and the RNA-induced silencing complex (RISC) slices complementary RNA and promotes RNA degradation (Borges and Martienssen 2015; Fukudome and Fukuhara 2017). Unexpected gene silencing is a disadvantage of protein production in biotechnology. Thus, studying RNA silencing is important not only for understanding plant growth and immunity, but also for applications such as protein production science.

Green fluorescent protein (GFP) is a commonly used reporter for RNA-silencing studies by agroinfiltration. For example, co-agroinfiltration of a GFP gene with the inverted-repeat (IR)-GFP sequence into *Nicotiana benthamiana* suppresses GFP fluorescence. Owing to its hairpin structure, an IR has the potential to function as an RDR-independent siRNA precursor (Zilberman et al. 2004; Mérai et al. 2005). In contrast, co-agroinfiltration with an RNA silencing suppressor, a protein that inhibits RNA silencing in plant viruses, enhances transient GFP fluorescence (Ruiz et al. 1998; Takeda et al. 2002)*.* Although GFP has been widely used as a standard reporter in molecular biology, its evaluation requires highly-sensitive cameras with excitation and emission filters. In addition, to quantify GFP, it is necessary to perform experiments such as western blotting, and when luciferase is used as a reporter, an additional substrate is required.

**Fig. 1:**
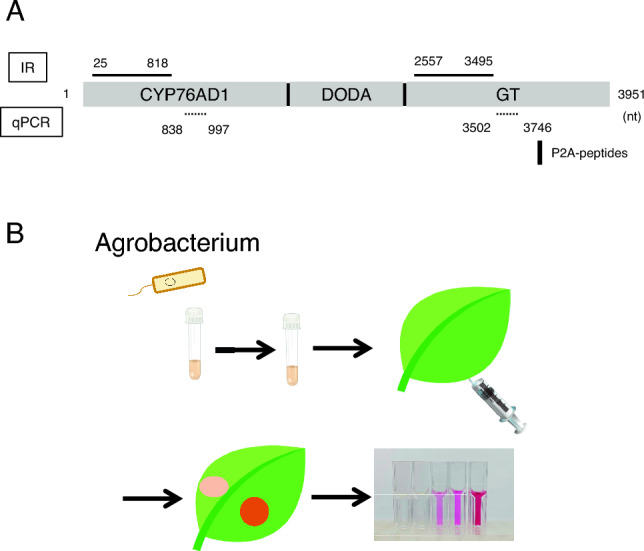
Agroinfiltration strategy with RUBY forward RNA silencing. **A** The gene structures of RUBY and inverted repeats (IR) against RUBY. RUBY is composed of P450 oxygenase CYP76AD1, l-DOPA 4,5-dioxygenase (DODA), and glycosyltransferase (GT), and it is united as a single open reading frame (ORF) by P2A-peptides, where the protein is cleaved by ribosomal skipping. Solid and dashed lines indicate the inverted-repeat and Reverse transcription quantitative PCR (RT-qPCR) target regions, respectively. **B** Schematic representation of agroinfiltration using RUBY. Cultured *Agrobacterium* transformed with plasmids containing RUBY and p19 were resuspended in agroinfiltration buffer and infiltrated into tobacco leaves with a syringe. The intensity of red color in the leaves was determined and quantified by absorbance

Recently, a novel reporter system was constructed as a RUBY system, which is based on the betalain biosynthesis machinery (Fig. 1A) (He et al. 2020). Betalain is a tyrosine-derived magenta pigment uniquely found in *Caryophyllales* (Strack et al. 2003). Betalain biosynthesis requires three key enzymes: P450 oxygenase CYP76AD1, l-3,4-dihydroxyphenylalanine (l-DOPA) 4,5-dioxygenase (DODA), and glycosyltransferase (GT). In the RUBY system, a single mRNA encoding these three enzymes linked by 2A peptides is transcribed, and then the three enzymes are translated into proteins that catalyze betalain (Fig. 1A). In transgenic *Arabidopsis thaliana* with RUBY derived from the cauliflower mosaic virus (CaMV) 35S promoter, the whole plant is colored pink by betalain, allowing RUBY expression to be recognized without any equipment or expensive reagents. Thus, RUBY is a promising marker system (He et al. 2020).

**Fig. 2:**
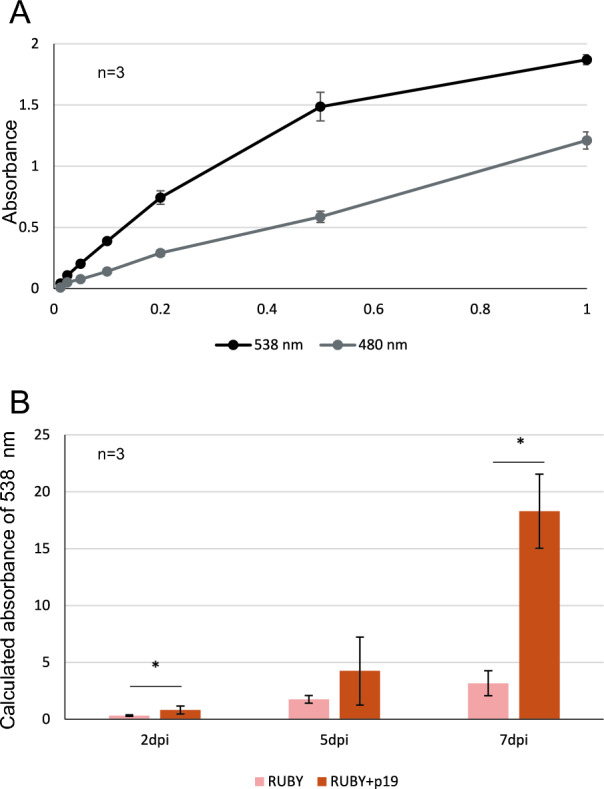
RNA silencing activities can be easily quantified by measuring the absorbance using the RUBY system. **A** Dilution series of leaf extracts for betalain measurements. Number 1 indicates the absorbance of the original extract. **B** Evaluation of RNA silencing by measuring absorbance of betalains. RUBY alone or a mixture of RUBY and p19 was agroinfiltrated; thereafter, the leaves were sampled and crushed to measure the absorbance. *dpi*: days post-infiltration. Error bars indicate standard deviations. n indicates biological replicates. Asterisks indicate statistically significant differences

In this study, we introduced the RUBY system into RNA silencing. First, we prepared two agrobacterial transformants with plasmids expressing RUBY and an RNA silencing suppressor, p19, both under the control of the 35S promoter. After the incubation of agrobacteria in liquid media and its pre-activation by acetosyringone, *Agrobacterium* was infiltrated into 4–6 week-old leaves (Fig. 1B). Betalain pigmentation was exhibited from 2 days post infiltration (dpi). Betalain can be quantified by measuring the absorbance at 538 nm and 480 nm wavelengths (Tomizawa et al. 2021). To test the correlation between betalain pigmentation and absorbance, a lysate was prepared from the infiltrated area at 7 dpi, and the intensity of betalains was measured by serial dilution to confirm the correlation (Fig. 2A). Next, to test the effect of the RNA silencing suppressor of RUBY pigmentation, RUBY was co-infiltrated with p19. The difference in red depth could be clearly distinguished between infiltration with RUBY alone and infiltration with both RUBY and p19 after 5 dpi. The absorbance indicated that five times more pigment was deposited in areas of leaves infiltrated with RUBY and p19 than in areas of leaves infiltrated with RUBY alone (Fig. 2B, Supplementary Fig. S1). These results reflect that RUBY itself, overexpressed by agroinfiltration, induces RNA silencing and that p19 suppresses the RNA silencing. It suggests that RUBY can be used to evaluate a suppressive ability of RNA silencing suppressors (Baulcombe 2004). Notably, even at 7 dpi, the betalain pigment did not fade, but rather the difference became more pronounced. When using protein reporters such as GFP, it is difficult to interpret the RNA silencing level after a number of days because the protein is degraded independently of the RNA-level silencing (Baulcombe 2004). On the other hand, biosynthesized betalains are stable and continue to accumulate; therefore, the longer the observation is continued, the more pronounced the difference in pigmentation becomes, and even very small differences in RNA silencing activity are likely to be easier to detect. Furthermore, betalains are a good reporter for continuous observation, as the color shade can be distinguished at a glance without leaf disruption. Thus, an RNA silencing assay with RUBY is a robust tool with the potential to detect overlooked differences.

To determine the optimal *Agrobacterium* concentration for the RUBY system, we infiltrated tobacco leaves with different concentrations of *Agrobacterium*. A clear difference in betalain absorbance between areas with and without p19 was observed at all concentrations tested (OD_600_ 0.125, 0.25, 0.5, and 1.0), with the most significant absorbance difference occurring at OD_600_ 0.5 or 1.0 (Supplementary Fig. S2B, C, E, F). Regarding the visual difference in betalain pigmentation on tobacco leaves, the contrast in color density between areas with and without p19 was easily noticeable at ODs ranging from 0.125 to 0.5. However, this color density difference became difficult to detect at an OD_600_ of 1.0 (Supplementary Fig. S2A, D). Therefore, we determined that an *Agrobacterium* concentration of OD_600_ 0.5 was the most appropriate for the RUBY-based RNA silencing quantification system. Additionally, we confirmed a strong correlation between the absorbance measurements at 538 nm and 480 nm, indicating that a single wavelength measurement could be sufficient (Supplementary Fig. S2B–F).

**Fig. 3:**
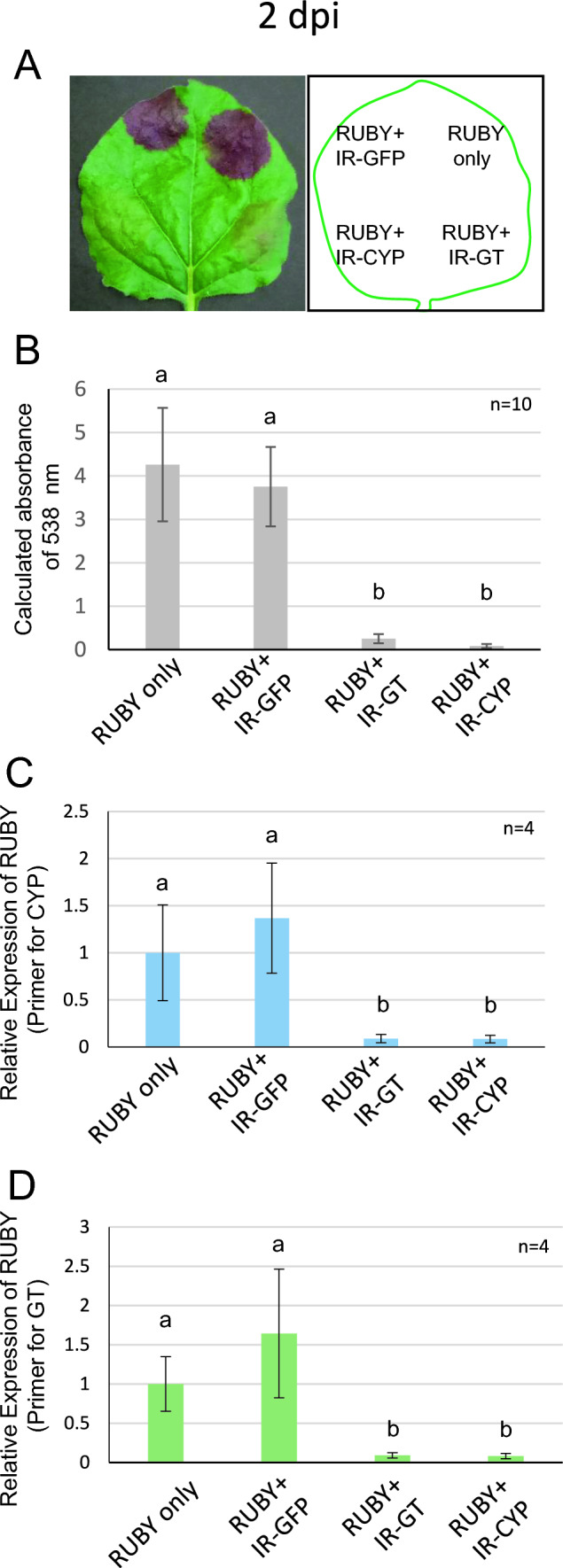
Inverted repeat-induced RNA silencing with the RUBY system. **A** A representative image of Tobacco leaf agroinfiltrated with RUBY alone, RUBY and IR-GFP, RUBY and IR-GT, or RUBY and IR-CYP. **B** Absorbance of extracts from the infiltrated regions indicated in **A**. **C–D** RT-qPCR for RUBY mRNA using the RNA extracted from the infiltrated region indicated in **A**. The primer set for detection of CYP was used in **C** and that of GT was used in **D**. Error bars indicate standard deviations. n indicates the number of biological replicates. Different alphabets represent statistically significant differences

Next, we constructed plasmids to transcribe IR-RNA targeting specific sequences within the RUBY gene. The open reading frame of RUBY is 4.0 kbp and longer than general reporter genes, such as GFP or Luciferase. Therefore, we designed two long IRs against CYP67AD1 (IR-CYP, 846 bp) and GT (IR-GT, 989 bp) in RUBY (Fig. 1A). Clear visual differences in betalain pigmentation were observed as early as 2 dpi, with leaf sections co-infiltrated with either IR vector (IR-CYP or IR-GT) exhibited less betalain coloration compared to those infiltrated with RUBY alone or with IR-GFP, a negative control IR (Fig. 3A). This reduction in pigmentation corresponded to absorbance values at 538 nm (4.26 ± 1.30 in RUBY alone, 3.75 ± 0.91 in RUBY + IR-GFP, 0.25 ± 0.11 in RUBY + IR-GT, and 0.08 ± 0. 05 in RUBY + IR-CYP; mean ± SD) and with RUBY mRNA levels (1.00 ± 0.51 in RUBY alone, 1.37 ± 0.59 in RUBY + IR-GFP, 0.09 ± 0.04 in RUBY + IR-GT, and 0.08 ± 0.04 in RUBY + IR-CYP; mean ± SD) at 2 dpi (Fig. 3B and C). The silencing activity of both IR constructs was further validated using specific primers designed in GT ORF region (Fig. 3D). The effective silencing by IR-GT and IR-CYP was also evident at both 5 and 7 dpi (Supplementary Fig. S3).

**Fig. 4:**
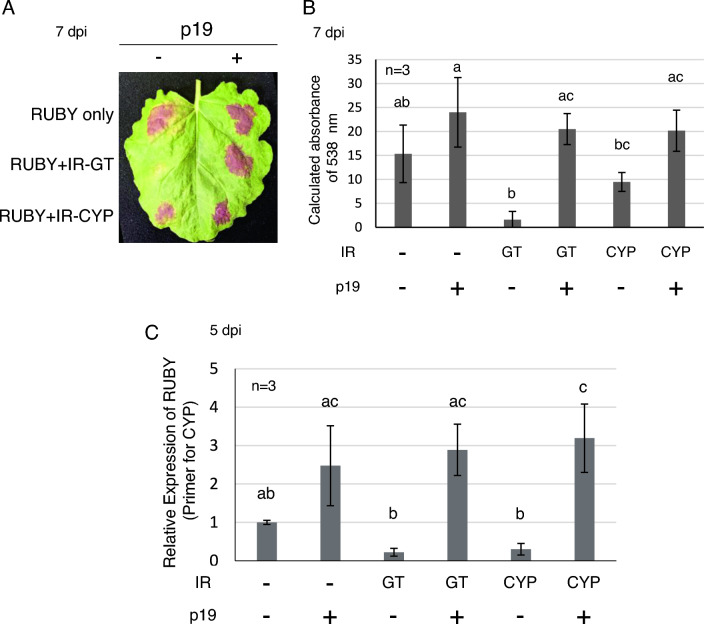
p19 strongly suppresses inverted repeat-induced RNA silencing. **A** Tobacco leaves agroinfiltrated with RUBY alone, RUBY and IR-GT, or RUBY and IR-CYP. The three locations on the right were simultaneously infiltrated with p19. **B** Absorbance of extracts from the infiltrated regions indicated in **A**. **C** RT-qPCR for RUBY mRNA using the RNA extracted from the infiltrated region indicated in **A**. The primer set for detection of CYP was used. Error bars indicate standard deviations. n indicates the number of biological replicates. Different alphabets represent statistically significant differences

In further experiments involving co-infiltration with both IR and p19, samples with IR constructs alone showed reduced pigmentation compared to the RUBY control, whereas co-infiltration with p19 showed intense red pigmentation, indicative of silencing suppression (Fig. 4A, Supplementary Fig. S4). Betalain content and RUBY mRNA levels in samples co-infiltrated with p19 showed little variation with or without IR constructs, demonstrating the ability of p19 to effectively counteract the silencing effects of IR (Fig. 4B, C). Although variations in pigmentation suppression by IR-CYP and IR-GT were observed, quantitative PCR (qPCR) analysis did not show significant differences between them (Figs. 3, 4). This suggests that the observed differences in betalain accumulation may be due to factors other than the direct silencing efficiency of the IR constructs on RUBY, possibly due to variations in betalain biosynthesis or other regulatory mechanisms. We consider that RUBY combined with other experiments such as qPCR would likely lead to more definitive conclusions. We also emphasize the importance of using healthy plants of uniform size grown under identical environmental conditions, as we have observed that plants in optimal health tend to have higher absorbance values.

Despite some considerations, the utility and simplicity of the RUBY system is very attractive. Unlike GFP, the RUBY system allows direct observation silencing activity through visual assessment of leaf pigmentation without the need for fluorescence detection. The results can be quantified quickly and easily by measuring absorbance, eliminating the need for Western blotting. If you want to compare a large number of experimental conditions, RUBY allows you to screen by appearance and absorbance and then validate the results by qPCR for the conditions of interest. The RUBY assay is also ideal for educational, training and research applications and provides a robust platform for RNA silencing studies. Consequently, the RNA silencing assay using RUBY is an excellent system that combines ease of use and dependability, and its widespread adoption is expected to advance RNA silencing research.

## Materials and methods

### Plant materials and growth conditions

*N. benthamiana* plants were grown with mixture of vermiculite and compost soil in pots in a room with a controlled environment under the following conditions: 50–100 μmol m^−2^ s^−1^, 16 h light and 8 h dark, 24 °C.

### Preparation of plasmids for agroinfiltration

We constructed KMd638 (p35S:dstrCYP76-t35S_pBIC, also described as IR-CYP) or KMd641 (p35S:dstrGluT-t35S_pBIC, also described as IR-GT) plasmids as follows: (1) we amplified two DNA fragments containing a truncated CYP76AD1 fragment (846 bp) using the KMol1290 and KMol1291 primers or a truncated GT fragment (989 bp) using the KMol1294 and KMol1295 primers from the KMd453 (35S:RUBY) plasmid (He et al. 2020); (2) we introduced these PCR products into the BamHI site of KMd612 (pBICdsGFP) (Takeda et al. 2002) using an XE cocktail (Liu et al. 2023) to create two intermediate plasmids; (3) we amplified two other DNA fragments using the KMol1292 and KMol1293 primers or a truncated GT fragment (989 bp) using the KMol1296 and KMol1297 primers from the 35S-RUBY plasmid; (4) we introduced these PCR products into the KpnI site of these intermediate plasmids using the XE cocktail. 35S:RUBY was a gift from Yunde Zhao (Addgene plasmid #160908; http://n2t.net/addgene:160908; RRID:Addgene 160908). Primer sequences used for PCR are described in Supplementary Table S1. An empty vector (pBIC35S) and p19 (pBIC35S:p19) was described previously (Takeda et al. 2002).

### *Agrobacterium*-mediated transient expression assay (agroinfiltration)

Binary plasmids were introduced into *A. tumefaciens* (*R. radiobacter*) strain GV3101 (MP90) by a Freeze–Thaw method (Weigel and Glazebrook, 2006) and then transformants were selected on Luria Broth (LB) agar medium containing 50 µg/mL each of Rifampicin, Gentamycin and Kanamycin (pBIC vector) or 50 µg/mL Spectinomycin (RUBY) at 28 °C for 2 or 3 days. Transformed *Agrobacterium* was inoculated into LB liquid medium containing antibiotics at 28 °C for 24 to 48 h, and then the culture solution was diluted with 50 volumes of the same LB medium and incubated for 16 to 24 h. Bacterial cells were harvested by centrifugation at 3000 rpm for 15 min and suspended in agroinfiltration buffer (10 mM MES-NaOH [pH 5.6] and 10 mM MgCl_2_). Bacterial cells were harvested by centrifugation, re-suspended in agroinfiltration buffer containing 150 µM acetosyringone to OD_600_ 0.5, incubated in the dark at 26 °C for 3 to 5 h, and infiltrated into the leaves of wild type *N. benthamiana* plants that were 4 to 6 weeks old. To determine the appropriate concentration of *Agrobacterium*, bacterial cells were suspended in agroinfiltration buffer containing acetosyringone, then adjusted to an OD_600_ of 1, and incubated for 3 h before being serially diluted (with the dilutions explicitly marked as OD_600_ 0.5, 0.25, and 0.125). The serially diluted *Agrobacterium* was infiltrated into tobacco leaves. When co-infiltrating two vectors, each vector was independently transformed into *Agrobacterium*, then adjusted to the appropriate OD_600_ and then mixed before infiltration.

### Betalain content assay

Agroinfiltrated leaves were punched using the lid of a 1.5/2.0 mL centrifuge tube. Samples were frozen in liquid nitrogen and pulverized. Distilled water was added to the frozen tubes and mixed by vortexing. After centrifugation at 13,000×*g* for 5 min, the pigment was eluted into the supernatant. Non-diluted supernatant or diluted supernatant were used to measure their absorbances at 538 nm and 480 nm. If the absorbance of the non-diluted solution was higher than 1.5, the absorbance of the non-diluted solution was estimated from the absorbance of the diluted sample. The blank was measured using distilled water, and the negative control was measured with lysate of the mock area separately. Tukey’s test was performed using R (R core team 2013).

### Reverse transcription quantitative PCR (RT-qPCR)

Total RNA was isolated from agroinfiltrated spots of *N. benthamiana* leaves using the Trireagent® following the manufacturer’s protocol (MilliporeSigma, Burlington, MA, USA), from which cDNAs were produced by ReverTra Ace® qPCR RT Master Mix with gDNA Remover (TOYOBO, Osaka, Japan). qPCR was performed using an Applied Biosystems Step One Plus Real-Time PCR System (Thermo Fisher Scientific, Waltham, MA, USA) with the KOD SYBR mix (TOYOBO). Primers for qPCR were designed using the Primer3Plus program (http://www.bioinformatics.nl/cgi-bin/primer3plus/primer3plus.cgi/); the primer sequences are shown in Table S1.

### Statistical analysis

Comparisons between the two groups were made with Student’s *T* test. To evaluate results of betalain content assay and RT-qPCR, One way analysis of variance (ANOVA) and Tukey’s test were performed using the R Statistical Software (ver.4.2.1) (R core team 2013) for multiple comparisons. Quantitative values were expressed as the mean ± SD.

### Supplementary Information

Below is the link to the electronic supplementary material.

Supplementary file1 (PDF 641 kb)

Supplementary file2 (XLSX 16 kb)

## Data Availability

Raw data that support the findings of this study are available from the corresponding author upon reasonable request.

## References

[CR1] Baulcombe D (2004). RNA silencing in plants. Nature.

[CR2] Borges F, Martienssen RA (2015). The expanding world of small RNAs in plants. Nat Rev Mol Cell Biol.

[CR3] Fukudome A, Fukuhara T (2017). Plant dicer-like proteins: double-stranded RNA-cleaving enzymes for small RNA biogenesis. J Plant Res.

[CR4] Gelvin SB (2003). *Agrobacterium*-mediated plant transformation: the biology behind the “gene-jockeying” tool. Microbiol Mol Biol Rev.

[CR5] He Y, Zhang T, Sun H, Zhan H, Zhao Y (2020). A reporter for noninvasively monitoring gene expression and plant transformation. Hortic Res.

[CR6] Johansen LK, Carrington JC (2001). Silencing on the spot. Induction and suppression of RNA silencing in the *Agrobacterium*-mediated transient expression system. Plant Physiol.

[CR7] Liu AY, Koga H, Goya C, Kitabatake M (2023). Quick and affordable DNA cloning by reconstitution of seamless ligation cloning extract using defined factors. Genes Cells.

[CR8] Llave C, Kasschau KD, Carrington JC (2000). Virus-encoded suppressor of posttranscriptional gene silencing targets a maintenance step in the silencing pathway. Proc Natl Acad Sci USA.

[CR9] Mérai Z, Kerényi Z, Molnár A, Barta E, Válóczi A, Bisztray G, Havelda Z, Burgyán J, Silhavy D (2005). Aureusvirus P14 is an efficient RNA silencing suppressor that binds double-stranded RNAs without size specificity. J Virol.

[CR10] R Core Team (2013) R: a language and environment for statistical computing

[CR11] Ruiz MT, Voinnet O, Baulcombe DC (1998). Initiation and maintenance of virus-induced gene silencing. Plant Cell.

[CR12] Strack D, Vogt T, Schliemann W (2003). Recent advances in betalain research. Phytochemistry.

[CR13] Takeda A, Sugiyama K, Nagano H, Mori M, Kaido M, Mise K, Tsuda S, Okuno T (2002). Identification of a novel RNA silencing suppressor, NSs protein of *Tomato spotted wilt virus*. FEBS Lett.

[CR14] Tomizawa E, Ohtomo S, Asai K, Ohta Y, Takiue Y, Hasumi A, Nishihara M, Nakatsuka T (2021). Additional betalain accumulation by genetic engineering leads to a novel flower color in lisianthus (*Eustoma grandiflorum*). Plant Biotechnol.

[CR15] Tzfira T, Citovsky V (2006). Agrobacterium-mediated genetic transformation of plants: biology and biotechnology. Curr Opin Biotechnol.

[CR16] Weigel D, Glazebrook J (2006). Transformation of *Agrobacterium* using the freeze-thaw method. Cold Spring Harb Protoc.

[CR17] Zilberman D, Cao X, Johansen LK, Xie Z, Carrington JC, Jacobsen SE (2004). Role of Arabidopsis ARGONAUTE4 in RNA-directed DNA methylation triggered by inverted repeats. Curr Biol.

