# Supplementary material for: Straightforward and affordable agroinfiltration with RUBY accelerates RNA silencing research
Source: Plant Mol Biol. 2024 May 19;114(3):61. doi: 10.1007/s11103-024-01463-8 (PMC11102880; doi:10.1007/s11103-024-01463-8)
Supplement: Supplementary file 1 — Supplementary file1 (PDF 641 kb) [file 11103_2024_1463_MOESM1_ESM.pdf]

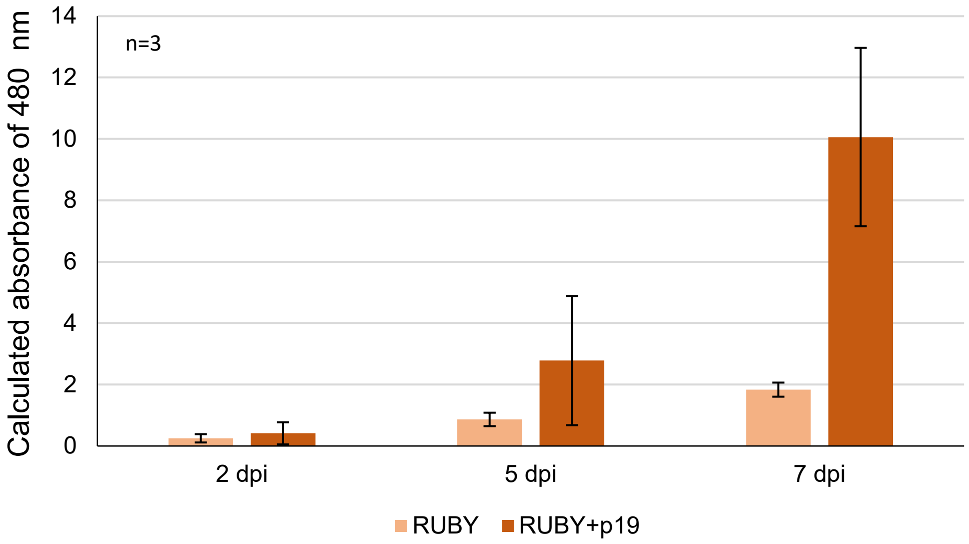

Supplementary Figure 1 Quantification of RNA silencing activities by measuring absorbance at 480 nm. RUBY alone or a mixture of RUBY and p19 was agroinfiltrated; thereafter, the leaves were punched out and crushed to measure absorbance. dpi: days post-infiltration. Error bars indicate standard deviation. n indicates the biological replicates.

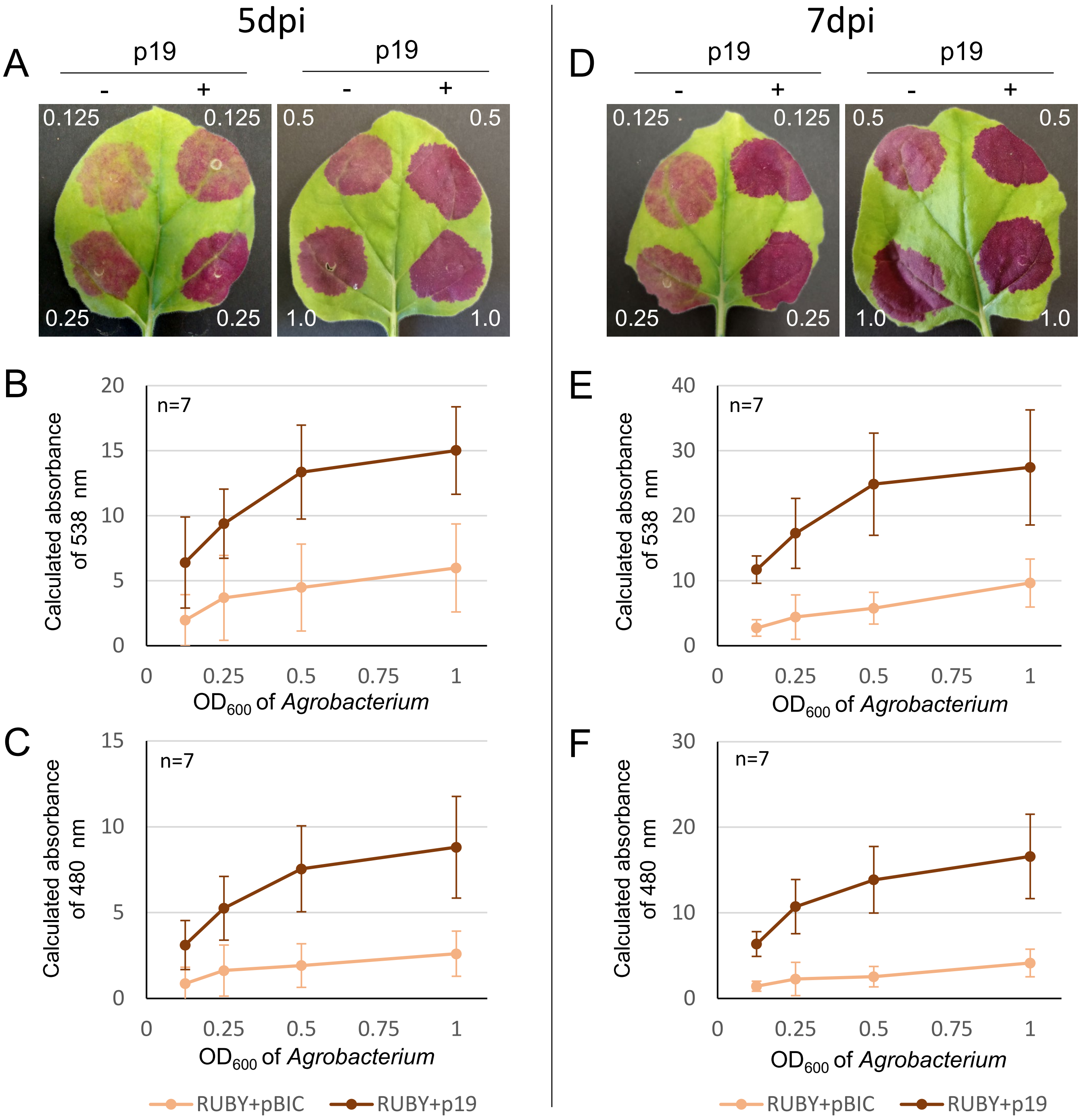

Supplementary Figure 2 Correlation between betalain accumulations and *Agrobacterium* infiltration at different ODs. (A-C) Quantification of betalain pigment at 5 days post infiltration (dpi). (A) Representative images of tobacco leaves at 5 dpi. The left side of the leaf was co-infiltrated with RUBY and empty vector, and the right side was co-infiltrated with RUBY and p19 vector. White letters indicate the OD_{600} of *Agrobacterium*. (B) Absorbance of 538 nm of the extracts from the infiltrated regions is shown in (A). (C) Absorbance of 480 nm of the extracts from the infiltrated regions is shown in (A). (D-F) Quantification of betalain pigment at 7 dpi. (D) Representative images of tobacco leaves at 7 dpi. The left side of the leaf was co-infiltrated with RUBY and empty vector, and the right side was co-infiltrated with RUBY and p19 vector. White letters indicate the OD_{600} of *Agrobacterium*. (E) Absorbance of 538 nm of the extracts from the infiltrated regions is shown in (D). (F) Absorbance of 480 nm of the extracts from the infiltrated regions is shown in (D). Error bars indicate standard deviation and n represents the number of biological replicates.

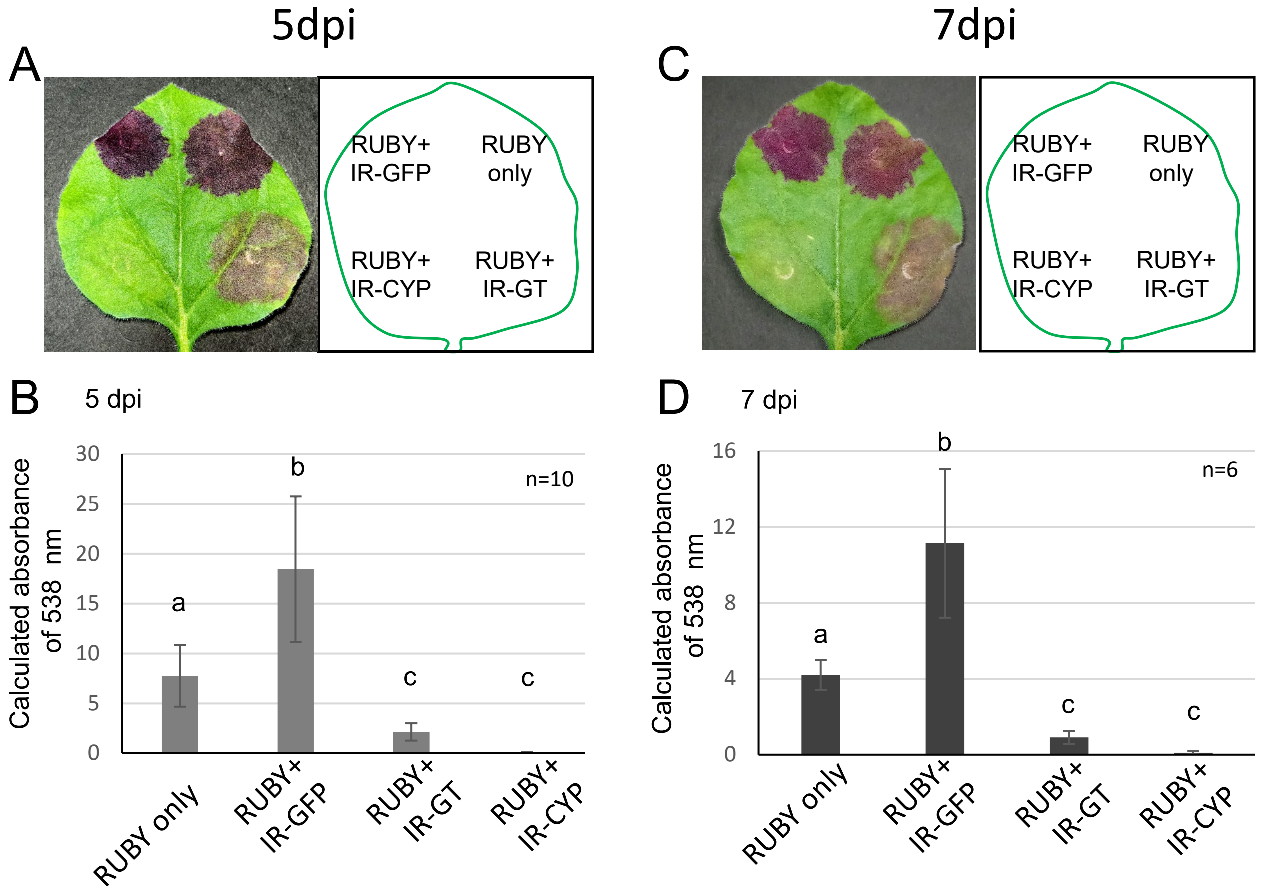

Supplementary Figure 3 Detection of inverted repeat-induced RNA silencing activity with the RUBY system at 5 or 7 dpi. (A) A representative image of Tobacco leaf agroinfiltrated with RUBY alone, RUBY and IR-GFP, RUBY and IR-GT, or RUBY and IR-CYP at 5 dpi. (B) Absorbance of extracts from the infiltrated regions indicated in (A). (C) A representative image of Tobacco leaf agroinfiltrated with RUBY alone, RUBY and IR-GFP, RUBY and IR-GT, or RUBY and IR-CYP at 7 dpi. (D) Absorbance of extracts from the infiltrated regions indicated in (C). Error bars indicate standard deviation and n represents the number of biological replicates. Different letters represent statistically significant differences.

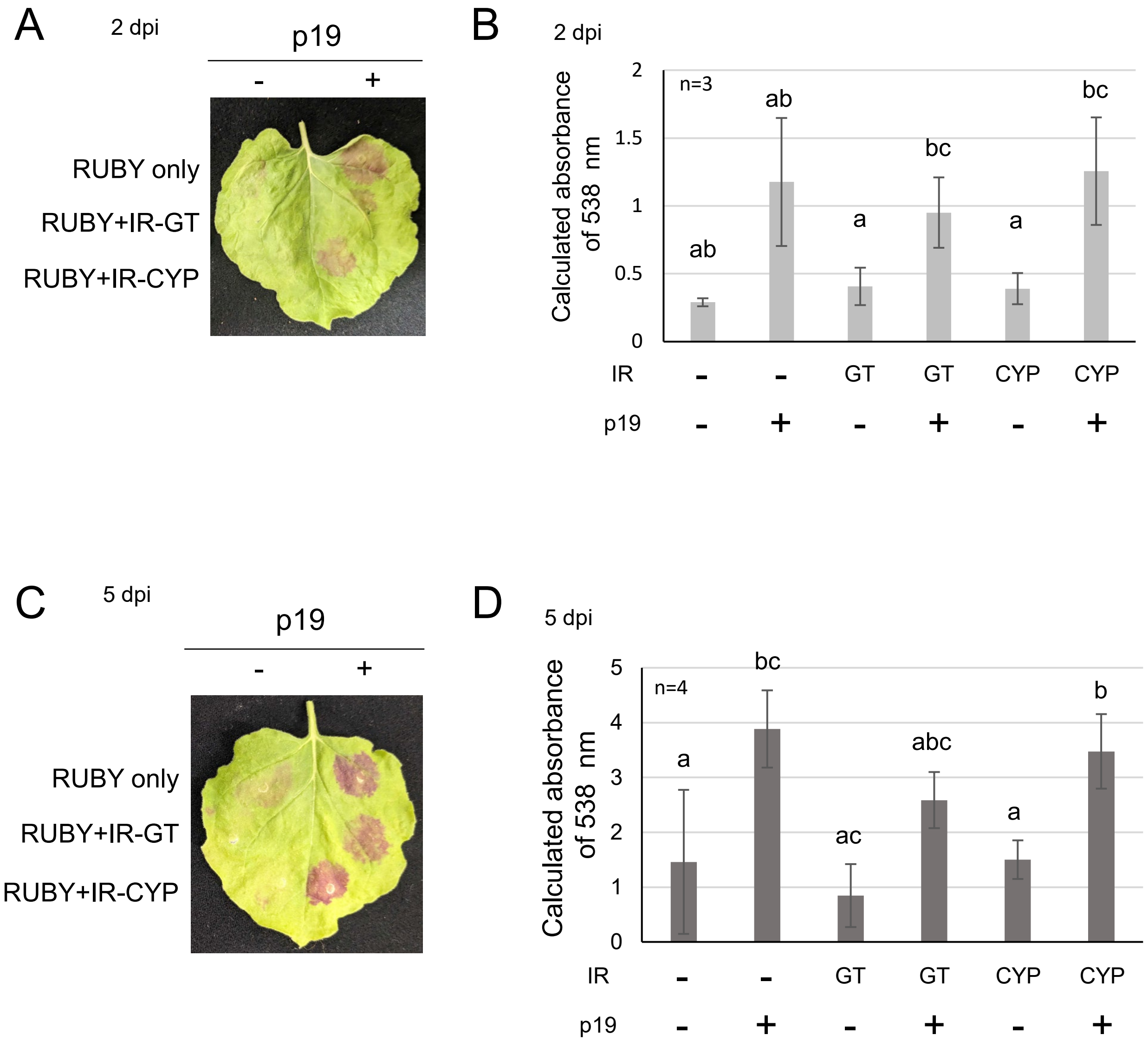

Supplementary Figure 4 Comparison of RNA silencing of co-infiltration of IR and p19 at 2 or 5 dpi. (A) Tobacco leaves were infiltrated with RUBY, RUBY + IR-GT, or RUBY + IR-CYP at 2 dpi, and three locations on the right were simultaneously infiltrated with p19. (B) Absorbance of the extracts from the infiltrated regions is shown in (A). (C) Tobacco leaves were infiltrated with RUBY, RUBY + IR-GT, or RUBY + IR-CYP at 5 dpi, and three locations on the right were simultaneously infiltrated with p19. (D) Absorbance of the extracts from the infiltrated regions is shown in (C). Error bars indicate standard deviation and n represents the number of biological replicates. Different letters represent statistically significant differences.
